# The Relationship between Social Support and Anxiety among Rural Older People in Elderly Caring Social Organizations: A Cross-Sectional Study

**DOI:** 10.3390/ijerph191811411

**Published:** 2022-09-10

**Authors:** Lanlan Zhao, Xin Zheng, Kai Ji, Zhengsheng Wang, Lingzhi Sang, Xuefei Chen, Ling Tang, Ying Zhu, Zhongliang Bai, Ren Chen

**Affiliations:** 1 School of Health Services Management, Anhui Medical University, Hefei 230032, China; 2 Suzhou Hospital Affiliated to Anhui Medical University, Suzhou 234000, China

**Keywords:** social support, anxiety, rural older people, elderly caring, social organizations

## Abstract

Background: Social support and anxiety have a major impact on later life quality in rural, older people in elderly caring social organizations (SOs). This study aimed to explore the relationship between social support and anxiety and their relevant influential factors among rural older people in elderly caring SOs in Anhui Province, China. Methods: This cross-sectional study was conducted through a multi-stage stratified cluster random sampling method. Independent *t*-tests, one-way ANOVA, Mann–Whitney U test, Kruskal–Wallis H test, and a generalized linear model were employed. Results: A significantly negative association between friends’ support and anxiety were found among rural older people in elderly caring SOs. Statistically significant relationships were found between social support and gender, marital status, education level, whether visited by relatives, and institutional satisfaction. Similarly, anxiety was associated with gender, institutional satisfaction, comorbid chronic diseases, and friends’ support. Conclusions: Social support from friends plays an important role in preventing and regulating anxiety among rural older people, especially those from elderly caring SOs. To reduce the occurrence and level of anxiety among rural elderly in elderly caring SOs, an effort should be given to strengthening social support, improving institutional satisfaction, and emphasizing comorbid chronic diseases.

## 1. Introduction

China is currently facing an aging challenge characterized by a large, rapidly aging population base. According to the seventh national census, the total population of China was 1.4 billion, with 264 million (18.7%) aged ≥ 60 years and 190 million (13.5%) aged ≥ 65, respectively [1]. As a result, the physical and mental health of older people and pension issues has attracted significant attention. Anxiety is a common mental disorder among elderly individuals, which can impair their social function, reduce the quality of life and life satisfaction, and even cause suicide [2,3,4]. Previous literature found that the prevalence of anxiety and anxiety symptoms in older adults ranged from 1.2% to 15% and 15% to 52.3%, respectively [5]. The prevalence of anxiety disorders varies greatly all over the world [6], and the current global prevalence of anxiety disorders was estimated to be 7.3%, ranging from 4.8% to 10.9% [7]. Therefore, anxiety disorders are increasingly regarded as a global public health problem, causing a huge economic burden [8]. While most studies on anxiety among the elderly were conducted in either community or clinical settings, few have been specifically devoted to elderly caring SOs. Social organizations refer to various organizations that are neither governmental organizations nor market organizations, also called third-party organizations [9].

In China, nursing homes and elderly apartments are operated by government organizations, SOs, or private investors that provide services for the elderly [10,11]. With the change in China’s family structure and people’s old-age care concepts, an increasing number of older people have opted to stay in elderly caring SOs [9,12]. The fourth sampling survey on the living conditions of the elderly in China in 2016 found that only 27.7% of the rural elderly self-reported as “good”, as opposed to the national average 32.8% [13]. Compared with urban elderly, rural elderly reported lower happiness and more depression and loneliness [14,15,16]. Therefore, more attention should be paid to the influencing factors of anxiety among rural elderly, especially in elderly caring SOs.

In recent years, most studies have demonstrated that various factors such as comorbid physical problems, isolation, bereavements, and living companions expose older people to relevant levels of anxiety [17,18,19]. Perceived social support was increasingly acknowledged as a significant coping mechanism affecting anxiety [20,21,22,23,24]. Social support refers to the support perceived by an individual from social network members [25]. In Italy, a study found that perceived emotional support was a negative predictor of symptoms of anxiety in older outpatients [26]. Furthermore, gender and social support were found to be the influential factors of anxiety [27,28,29]. Social support was found to have protective effects that reduced anxiety in older adults [27], and women were found to report higher levels of anxiety than men among elderly Arab people [29].

Although many studies have investigated the relationship between social support and anxiety in China, there is a lack of research on Chinese elderly in elderly caring SOs. Considering the Chinese aging problem, this study focused on the rural older people from the elderly caring SOs in Anhui Province. It attempted to explore the relationship between social support (family support, friends’ support, and significant others support) and anxiety and their relevant influential factors. Eventually, this study found that anxiety was significantly negatively correlated with social support from friends, and provided a reference for reducing anxiety levels and improving social support levels of the rural elderly in elderly caring SOs.

## 2. Methods

### Study Design and Data Collection

This cross-sectional study was conducted in November and December 2019 in Anhui Province, China. Participants were selected through a multi-stage stratified cluster random sampling method [9,30], and a structured questionnaire was used. With the help of local managers, face-to-face interviews were conducted by a skilled and trained survey team from Anhui Medical University. First, 6 cities (Anqing, Chizhou, Huainan, Luan, Suzhou, and Fuyang) from Anhui Province were chosen according to their geographical location and aging levels. Second, all counties or districts included in the 6 cities were regarded as the study sample area, and 15 districts or counties were selected. Subsequently, we obtained a directory of elderly caring social organizations from the local civil affairs system and randomly selected half of them. In the selected organizations, 10% of the rural older people will be randomly selected. If the number of rural older people in the organization is less than 5 people, all will be conducted. If the number of rural elderly in the organization is 5–49, then 5 people will be randomly selected. The maximum number of rural older people selected from one organization was 10. The inclusion criteria of the participants included residents in elderly caring SOs, aged ≥ 60 years, rural household registration, and willingness to participate in the study. Those who were reluctant to participate in the study, unable to communicate effectively with investigators, or unable to understand due to organic mental problems (visual problems, auditory problems, dementia) were excluded. Finally, a total of 822 valid questionnaires were analyzed.

## 3. Measure

### 3.1. Sociodemographic Characteristics

The sociodemographic characteristics contained 8 questions, including gender (male, female), age (≤74, 75–89, and ≥90 years), education level (unschooled, primary school, middle school, high school, and above), length of stay (≤12, 13–24, 25–36, ≥37 months), whether visited by relatives (yes, no), self-reported institutional satisfaction (satisfied and dissatisfied), marital status, and comorbid chronic diseases. The marital status was divided into married (having spouses), single, and other (widowed or divorced). As for comorbid chronic diseases, participants who have been diagnosed with two or more chronic diseases (diabetes, heart disease, malignancies, cancer, and so on) were defined as yes, while those who were diagnosed with one or no chronic disease were defined as no [31,32].

### 3.2. Social Support

The Chinese version of the Multidimensional Scale of Perceived Social Support (MSPSS) was used to assess the level of social support [33]. The scale is divided into 3 subscales (family, friends, and significant others) with 12 items in total. In this study, the score of each item was calculated on a 7-point scale (1 = very strongly disagree to 7 = very strongly agree). The total score of MSPSS was the sum of all items and ranged from 12 to 84. A high score indicates that participants perceived higher social support. The MSPSS has been widely used in Chinese samples, with Cronbach’s alpha being 0.863 [34]. In this study, Cronbach’s alpha of the whole scale, family support subscale, friends’ support subscale, and significant others support subscale were 0.978, 0.985, 0.971, and 0.962, respectively.

### 3.3. Anxiety

The Generalized Anxiety Disorder 7-item Scale (GAD-7) was used to screen anxiety and assess changes in the severity of anxiety symptoms in the individuals [35,36]. The scale contains 7 items raged on a 4-point scale by considering the last 2 weeks. The answers for each item were scored as 0 (not at all), 1 (several days), 2 (more than half the days), and 3 (nearly every day) [35]. The total score ranged from 0 to 21, with higher scores indicating greater anxiety. A study performed in 2006 reported excellent internal consistency (Cronbach α = 0.92) and good reliability of GAD-7 [35]. Additionally, Cronbach’s alpha of the scale in this study was 0.874.

### 3.4. Statistical Analysis

The data were analyzed by SPSS 22.0 (IBM Corp., Armonk, NY, USA) and *p* < 0.05 was deemed significant in all tests. First, percentages, mean, and standard deviation were used to describe the sociodemographic characteristics, social support scores, and anxiety scores. Second, an exploratory analysis was used to test the scale data. If the data were normally distributed or homogenized, independent *t*-tests and one-way ANOVA were performed for binary variables (gender, whether visited by relatives, self-reported institutional satisfaction, comorbid chronic diseases) and categorical variables (age, marital status, education level, length of stay), respectively. Otherwise, the Mann–Whitney U test and the Kruskal–Wallis H test were performed. Finally, a generalized linear model was used for multivariate analysis.

## 4. Results

### 4.1. Descriptive Analysis

Of the 822 rural older people in organizations who were investigated, 66.20% were males and 33.80% were females. The average age of all respondents was 77.64 ± 7.78 years (age range: 60–104 years), and most were widowed or divorced (47.10%). The majority of the participants were unschooled (64.10%), 47.30% had been staying in elderly caring SOs for more than 3 years (36 months), and 73.70% were visited by their relatives. Furthermore, a large proportion of participants were satisfied with their organization (96.00%) and had not been suffering from comorbid chronic diseases (74.70%) (Table 1). 

### 4.2. Associations between Anxiety and Social Support and Sociodemographic Variables among Participants

The results revealed that social support, family support, friends’ support, and significant others’ support were all significantly correlated with gender, age, marital status, length of stay, whether visited by relatives, and self-reported institutional satisfaction. Friends’ support was significantly correlated with education level. Additionally, among all demographic variables, only gender, educational level, self-reported institutional satisfaction, and comorbid chronic diseases were significantly correlated with anxiety (Table 2).

### 4.3. Analysis Results of the Generalized Linear Model 

As shown in Table 3, gender, marital status, education level, whether visited by relatives, and self-reported institutional satisfaction were significant influential factors of social support. In addition, gender, self-reported institutional satisfaction, comorbid chronic diseases, and social support from friends were significant influential factors of anxiety among rural older people in elderly caring SOs (Table 4). In addition, females (β = 1.191, *p* < 0.001) were likely to report higher scores in anxiety than males. Participants who were satisfied with their organization (β = −1.583, *p* = 0.003) were likely to report lower scores in anxiety, and participants with comorbid chronic diseases (β = 0.657, *p* = 0.007) were likely to have higher anxiety than those without. In contrast, participants with lower levels of friends’ support (β = −0.079, *p* = 0.016) were likely to have higher anxiety.

## 5. Discussion

Anxiety has seriously impaired the health of older people [37,38]. The elderly in rural areas are particularly vulnerable, as they are disadvantaged by their lower financial position and socioeconomic status [39,40]. In elderly care services, the elderly caring SOs play an increasingly important role in planning and developing relevant services for the elderly [11,41]. This study aimed to explore the relationship between social support and anxiety and their relevant influential factors among rural older people in elderly caring SOs in Anhui Province, China. 

This study determined that gender, marital status, education level, whether visited by relatives, and self-reported institutional satisfaction were significantly related to social support. Among older people, males perceived less social support than females, which was similar to a study in Australia [42]. It may be related to the fact that females talk more easily about their inner pressures and troubles than males, and that they actively sought and accepted help from elderly care workers, friends, and relatives. 

The rural elderly who married demonstrated a higher level of social support than those who were single. A study conducted in Macao, Hong Kong, and Guangzhou reported a similar result [43]. Spouses create a sense of love and affection for older people, which plays an important role in terms of social support (such as emotional support) and stress relief [44,45,46]. However, those unmarried (single) rural older people who had no spouse or children, thus may receive less support and assistance from their family than those who were married. This study also found that the level of social support was higher in the rural elderly with primary school than those unschooled, which was similar to the findings reported in a previous study [47]. A possible reason could be that the rural older people with primary school may have better communication skills, higher socioeconomic status, and higher level of awareness to cope with difficulties through the help and support of others than those who are unschooled. 

Never being visited by relatives may result in rural elderly perceiving low social support from their family, which reflects the findings of a study revealing a correlation between the frequency of family visits and social support in elderly caring SOs [48]. Relatives are mainly the children of rural older people, who are the biggest source of economic, spiritual, and life support for the rural elderly in organizations. Those people who were often visited may obtain more material help, spiritual care, and higher social support. The elderly with higher self-reported institutional satisfaction perceived higher social support. A possible reason could be that rural older people with higher self-reported institutional satisfaction generally were in a better mood, and may have more trust and dependence on organization staff. Thus, their awareness of maintaining health through the help and support of the organization’s staff was stronger than those who were dissatisfied, which ultimately increased their level of social support.

In this study, the results suggested a significant relationship between anxiety, gender, self-reported institutional satisfaction, comorbid chronic diseases, and friends’ support. Under the background of a negative correlation between social support and anxiety, although women perceived higher levels of social support than men, women still reported higher levels of anxiety than men in this study. This result was consistent with prior findings [49,50]. Females were more likely to have anxiety compared to males, which may be related to genetic, biological, and sociopsychological factors [39]. Males are generally more active than females in social activities, where they can release their emotions. Females are easier to establish good communication and relationship with the outside world, which could improve their level of social support from friends. However, the positive coping ability of women has been reported to be lower than that of men [51]. Facing emergencies, older women were more likely to experience greater stress and anxiety than older men, which was associated with unstable temperament, weak stress resistance, and overthinking. 

Self-reported institutional satisfaction refers to the subjective evaluation of the elderly on the overall satisfaction level of the elderly caring SOs, mainly including quality of the environment, service, and life [11]. The present study noted that higher self-reported institutional satisfaction was correlated with a lower risk of anxiety among rural elderly in elderly caring SOs. Rural elderly with a lower self-reported institutional satisfaction were more likely to have a lower quality of life, service, and environment. In addition, lower quality of life may result in anxiety [52]. Similar to our results, an earlier study found that the higher the level of self-reported institutional satisfaction, the lower the level of anxiety [11]. 

Our study found that rural older people with comorbid chronic diseases had a higher chance of experiencing anxiety than those with no or only one chronic disease. Available literature indicates that comorbid chronic diseases were positively associated with anxiety [32,49,53]. Chronic diseases are common among older people, which affect their quality of life [32]. Those rural elderly with comorbid chronic diseases bore a great economic burden and suffered both physically and psychologically.

Additionally, our study found that there was a significantly negative correlation between friends’ support and anxiety among the rural elderly in elderly caring SOs, while the correlation between anxiety and family support and significant others’ support was not significant. This was consistent with previous findings, which indicated that social support had a protective effect against anxiety in the elderly [27,28]. Specifically, the level of anxiety was lower among those with a higher level of friends’ support. A possible reason may be the specific sample characteristics of the study. Most rural older people in this study were either divorced or widowed and satisfied with the elderly caring SOs. Thus their perceived social support from family and significant others was little. The rural older people who lived in elderly caring SOs mostly had contact with their friends (most were the elderly or elderly care workers) around them., As such, the social support they received from friends was more timely and daily than that from family and significant others. Therefore, it was more important for the rural elderly to experience and adequately use the social support from their friends in elderly caring SOs.

Based on our results, the improvement of social support (especially friends’ support) and self-reported institutional satisfaction plays an essential role in preventing and regulating anxiety among rural older people in elderly caring SOs. More importantly, health education should be promoted to alleviate the anxiety symptoms of chronic diseases in the rural elderly. We proposed social support from family, social organizations, care providers, fellow residents, and organization’s staff be integrated into the interventions of elderly caring SOs to increase social support and self-reported institutional satisfaction of rural elderly living in organizations. After admission, the organization’s staff should try to understand the basic situation, past life, and needs of rural elderly through conversations with them and their relatives to provide better-personalized services. Moreover, more institution-based collective activities, nursing service activities, and lectures on psychology and health should be conducted to increase social opportunities for rural elderly and improve their physical health and mental well-being. In addition, family visits should be regarded as a basic service item in elderly caring SOs. In the context of COVID-19, we suggested the organizations make full use of information communication technology and provide an online, face-to-face opportunity for older people. Information communication technology provides effective methods to help older people get in touch with their relatives (especially their children and grandchildren), friends, and significant others via digital interactions, regardless of temporal and geographical boundaries [54]. Connections could promote the level of social support among the elderly. Establishing a high functioning characteristic elderly care service system requires the cooperation of the family, society, government, social organizations, and other aspects of society. Contacts inside and outside the elderly caring SOs may assist in increasing social support for rural elderly, improving their mental health, and promoting healthy aging in the real sense. 

However, this study involved some limitations. First, this study was cross-sectional, so causal relationships could not be determined and further study is needed to verify our conclusions. Second, the data were collected by self-report measures, exposing the data collection to recall and reporting bias. In addition, there were some gaps in the collected data. Thirdly, the subjects of this study were all from Anhui Province, and the results may not be extended to other regions in China.

## 6. Conclusions

This study revealed a negative relationship between social support (from friends) and anxiety among rural older people in elderly caring SOs in Anhui, China. The elderly with higher levels of social support from friends were more likely to have lower levels of anxiety. Strengthening social support, improving self-reported institutional satisfaction, and alleviating comorbid chronic diseases play a positive role in reducing the level of anxiety. 

## Figures and Tables

**Table 1:** Descriptive results of the participants (*n* = 822).

Variables	*n* (%), Mean ± SD
**Gender**	
Male	544 (66.20)
Female	278 (33.80)
**Age (years)**	
≤74	333 (40.50)
75–89	445 (54.10)
≥90	44 (5.40)
**Marital status**	
Married	125 (15.20)
Single	310 (37.70)
Other	387 (47.10)
**Education level**	
Unschooled	527 (64.10)
Primary school	216 (26.30)
Middle school	68 (8.30)
High school and above	11 (1.30)
**Length of stay (months)**	
≤12	191 (23.20)
13–24	152 (18.50)
25–36	90 (10.90)
≥37	389 (47.30)
**Whether visited by relatives**	
No	216 (26.30)
Yes	606 (73.70)
**Self-reported institutional satisfaction**	
Dissatisfied	33 (4.00)
Satisfied	789 (96.00)
**Comorbid chronic diseases**	
No	614 (74.70)
Yes	208 (25.30)
**Anxiety**	1.91 ± 3.12
**Social support**	57.79 ± 16.64
Family support	18.96 ± 7.19
Friends’ support	18.92 ± 6.01
Significant others support	19.90 ± 5.91

**Table 2:** Associations between social support and anxiety and sociodemographic variables among participants. (M ± SD).

	Social Support (Total)	Family Support	Friends’ Support	Significant Others’ Support	Anxiety
**Gender**					
Male	54.54 ± 16.78	17.13 ± 7.36	18.30 ± 6.13	19.11 ± 5.99	1.60 ± 2.80
Female	64.14 ± 14.41	22.54 ± 5.23	20.14 ± 5.57	21.46 ± 5.44	2.51 ± 3.61
t/F/z/H	−8.534	10.641	4.320	5.727	3.240
*p*	<0.001 **	<0.001 **	<0.001 **	<0.001 **	0.001 *
**Age (years)**					
≤74	54.95 ± 16.38	17.09 ± 7.53	18.43 ± 6.22	19.42 ± 6.08	2.12 ± 3.27
75–89	59.30 ± 16.26	20.06 ± 6.59	19.14 ± 5.79	20.11 ± 5.71	1.78 ± 3.01
≥90	64.00 ± 18.93	22.02 ± 7.08	20.45 ± 6.33	21.52 ± 6.33	1.66 ± 3.10
t/F/z/H	9.974	44.028	7.154	9.185	5.844
*p*	<0.001 **	<0.001 **	0.028 *	0.010 *	0.054
**Marital status**					
Married	64.06 ± 15.85	22.26 ± 5.79	20.22 ± 5.70	21.58 ± 5.72	2.22 ± 3.47
Single	48.73 ± 15.90	14.15 ± 6.73	17.03 ± 6.41	17.55 ± 6.13	1.72 ± 2.86
Other	63.02 ± 14.13	21.75 ± 5.74	20.02 ± 5.36	21.24 ± 5.16	1.96 ± 3.21
t/F/z/H	89.982	244.358	47.386	80.531	1.168
*p*	<0.001 **	<0.001 **	<0.001 **	<0.001 **	0.558
**Education level**					
Unschooled	57.02 ± 16.77	18.90 ± 7.15	18.49 ± 6.12	19.62 ± 5.96	1.84 ± 3.14
Primary school	58.69 ± 16.88	18.81 ± 7.51	19.53 ± 5.85	20.34 ± 5.80	2.20 ± 3.31
Middle school	60.54 ± 15.31	19.59 ± 6.91	20.25 ± 5.52	20.71 ± 6.00	1.72 ± 2.49
High school and above	60.09 ± 11.44	20.73 ± 2.72	19.36 ± 4.72	20.00 ± 4.88	0.45 ± 1.51
t/F/z/H	1.282	0.751	8.295	4.373	8.378
*p*	0.279	0.861	0.040 *	0.224	0.039 *
**Length of stay (months)**					
≤12	61.68 ± 14.69	21.12 ± 6.15	19.71 ± 5.38	20.85 ± 5.25	1.79 ± 3.36
13–24	62.69 ± 17.56	21.43 ± 6.84	20.01 ± 6.07	21.24 ± 6.13	2.14 ± 3.35
25–36	60.59 ± 15.19	20.18 ± 6.56	19.82 ± 5.45	20.59 ± 5.68	2.08 ± 3.14
≥37	53.31 ± 16.38	16.66 ± 7.23	17.90 ± 6.25	18.76 ± 5.99	1.84 ± 2.91
t/F/z/H	19.316	91.277	23.249	33.846	3.085
*p*	<0.001 **	<0.001 **	<0.001 **	<0.001 **	0.379
**Whether visited by relatives**					
No	45.06 ± 15.76	11.55 ± 6.67	16.57 ± 6.54	16.93 ± 6.39	1.84 ± 3.05
Yes	62.33 ± 14.54	21.60 ± 5.26	19.76 ± 5.58	20.96 ± 5.35	1.93 ± 3.15
t/F/z/H	−14.719	16.524	6.614	8.197	−0.315
*p*	<0.001 **	<0.001 **	<0.001 **	<0.001 **	0.753
**Self-reported institutional satisfaction**					
Dissatisfied	46.52 ±18.87	15.18 ± 7.97	15.48 ± 6.85	15.85 ± 6.59	4.03 ± 4.79
Satisfied	58.26 ± 16.38	19.12 ± 7.11	19.07 ± 5.93	20.07 ± 5.83	1.82 ± 3.01
t/F/z/H	−4.009	2.928	2.988	3.727	−2.985
*p*	<0.001 **	0.003 *	0.003 *	<0.001 **	0.003 *
**Comorbid chronic diseases**					
No	58.07 ± 16.58	19.16 ± 7.14	18.95 ± 5.98	19.97 ± 5.95	1.71 ± 2.93
Yes	56.95 ± 16.82	18.38 ± 7.31	18.85 ± 6.11	19.72 ± 5.82	2.50 ± 3.57
t/F/z/H	0.842	−1.294	−0.280	−0.677	2.971
*p*	0.400	0.196	0.779	0.498	0.003 *

M, mean; SD, standard deviation; * *p* < 0.05; ** *p* < 0.001.

**Table 3:** Analysis results of the generalized linear model for the relationship between social support and sociodemographic variables among participants.

Variable	Social Support		
	Regression Coefficient (S.E.)	95% CI	*p*
Constant	42.178 (3.215)	35.877, 48.478	<0.001 **
**Gender**			
Male (ref)			
Female	2.698 (1.290)	0.171, 5.226	0.036 *
**Age (years)**			
≤74 (ref)			
75–89	−0.708 (1.088)	−2.841, 1.424	0.515
≥90	1.314(2.406)	−3.401, 6.030	0.585
**Marital status**			
Married (ref)			
Single	−8.215 (1.735)	−11.615, −4.816	<0.001 **
Other	−1.496 (1.504)	−4.443, 1.451	0.320
**Education level**			
Unschooled (ref)			
Primary school	2.756 (1.186)	0.432, 5.080	0.020 *
Middle school	2.081 (1.884)	−1.610, 5.773	0.269
High school and above	−0.800 (4.349)	−9.324, 7.725	0.854
**Length of stay (months)**			
≤12 (ref)			
13–24	0.260 (1.538)	−2.754, 3.274	0.866
25–36	−0.247 (1.805)	−3.785, 3.291	0.891
≥37	−1.628 (1.340)	−4.255, 0.998	0.224
**Whether visited by relatives**			
No (ref)			
Yes	11.650 (1.317)	9.068, 14.232	<0.001 **
**Self-reported institutional satisfaction**			
Dissatisfied (ref)			
Satisfied	10.808 (2.526)	5.858, 15.757	<0.001 **
**Comorbid chronic diseases**			
No (ref)			
Yes	−1.130 (1.139)	−3.362, 1.102	0.321

S.E. Standard error; CI, confidence interval; ref, reference; * *p* < 0.05; ** *p* < 0.001.

**Table 4:** Analysis results of the generalized linear model for the relationship between anxiety and three subscales of social support and sociodemographic variables among participants.

Variable	Anxiety		
	Regression Coefficient (S.E.)	95% CI	*p*
Constant	4.859 (0.751)	3.388, 6.331	<0.001 **
**Gender**			
Male (ref)			
Female	1.191 (0.275)	0.651, 1.730	<0.001 **
**Age (years)**			
≤74 (ref)			
75–89	−0.327 (0.232)	−0.780, 0.127	0.158
≥90	−0.527(0.511)	−1.528, 0.474	0.302
**Marital status**			
Married (ref)			
Single	−0.416 (0.376)	−1.153, 0.322	0.269
Other	−0.375 (0.320)	−1.002, 0.251	0.240
**Education level**			
Unschooled (ref)			
Primary school	0.489 (0.253)	−0.006, 0.984	0.053
Middle school	0.076 (0.401)	−0.710, 0.862	0.849
High school and above	−0.982 (0.923)	−2.791, 0.826	0.287
**Length of stay (months)**			
≤12 (ref)			
13–24	0.355 (0.326)	−0.284, 0.995	0.276
25–36	0.252 (0.383)	−0.499, 1.003	0.511
≥37	0.121 (0.285)	−0.437, 0.679	0.671
**Whether visited by relatives**			
No (ref)			
Yes	0.375 (0.318)	−0.247, 0.998	0.237
**Self-reported institutional satisfaction**			
Dissatisfied (ref)			
Satisfied	−1.583 (0.542)	−2.645, −0.521	0.003 *
**Comorbid chronic diseases**			
No (ref)			
Yes	0.657 (0.242)	0.182, 1.131	0.007 *
**Family support**	−0.040 (0.023)	−0.085, 0.004	0.076
**Friends’ support**	−0.079 (0.033)	−0.144, −0.015	0.016 *
**Significant others’ support**	0.013 (0.035)	−0.056, 0.082	0.715

S.E. Standard error; CI, confidence interval; ref, reference; * *p* < 0.05; ** *p* < 0.001.

## Data Availability

The data are available from the corresponding author on reasonable request.
